# Nitrous Oxide Sourcing, Use and Harms: Insights From Australians Who Use Ecstasy/MDMA and Other Illicit Stimulants

**DOI:** 10.1111/dar.70032

**Published:** 2025-09-03

**Authors:** Jodie Grigg, Simon Lenton, Amy Peacock, Jessamine Soderstrom, Cate King, Natalie Thomas, Rachel Sutherland

**Affiliations:** ^1^ National Drug Research Institute and enAble Institute Curtin University Perth Australia; ^2^ National Drug and Alcohol Research Centre UNSW Sydney Sydney Australia; ^3^ School of Psychological Sciences University of Tasmania Hobart Australia; ^4^ Emergency Department Royal Perth Hospital Perth Australia; ^5^ Institute for Social Science Research University of Queensland Brisbane Australia

**Keywords:** drug monitoring, harm reduction, neurologic symptoms, nitrous oxide, Vitamin B12 deficiency

## Abstract

**Introduction:**

Increasing harms associated with nitrous oxide use have raised concerns, but limited evidence exists to inform harm reduction. This study aimed to identify how nitrous oxide is sourced, the products/forms used, awareness of health risks, engagement in harm reduction and experience of neurological symptoms.

**Methods:**

Data were collected via annual interviews (2021–2023) with cross‐sectional convenience samples of Australians who regularly used ecstasy/MDMA and/or other illicit stimulants and reported past 6‐month use of nitrous oxide (2021 *n* = 379; 2022 *n* = 315; 2023 *n* = 284).

**Results:**

The most commonly reported source of nitrous oxide in the past 6 months was convenience stores, followed by 24/7 delivery services. Sourcing from these retailers was also associated with heavier use. Reported use of larger cylinders (> 8 g) increased from 6% in 2021 to 26% in 2023. While most respondents demonstrated awareness of neurological risk (e.g., brain damage 63%; nerve damage 20%), only one‐fifth identified the risk of Vitamin B12 depletion and 17% were unaware of any risks. Almost one‐third (30%) reported limiting use per session, 36% limited frequency and 36% took no precautions. Reports of neurological symptoms rose from 5% in 2021 to 14% in 2023 among cross‐sectional samples, with few (*n* ≤ 5) receiving treatment.

**Discussion and Conclusions:**

Findings suggest increasing use of larger cylinders, alongside increasing neurological harms. Given the evolving regulatory and risk environment, close surveillance of usage and market trends is needed. The findings reinforce the need for balanced regulation and targeted education of retailers, clinicians and consumers to reduce harmful patterns of use and encourage early intervention.

**Summary**

- Between the 2021 and 2023 cross‐sectional samples, there were increasing reports of use of larger nitrous oxide cylinders (> 8 g), alongside increasing reports of persistent neurological symptoms (e.g., tingling).
- Sourcing from corner/convenience stores and 24/7 delivery services was associated with higher frequency and higher quantity nitrous oxide use.
- While most participants demonstrated awareness of a neurological risk associated with nitrous oxide use (e.g., brain damage 63%; nerve damage 20%) in 2021, only one‐fifth identified a risk of Vitamin B12 depletion, the cause of most adverse neurological effects.
- The findings highlight the need for close surveillance of usage and market trends, along with balanced regulation and targeted education for retailers, clinicians and consumers.

## 1 Introduction

Nitrous oxide is a dissociative anaesthetic used for sedation in clinical settings and for food and beverage preparation in hospitality settings [1]. It also has a lengthy history as an intoxicant due to its euphoric and psychedelic effects when inhaled [2] and has been growing in popularity internationally [1, 2, 3]. In Australia, evidence suggested a strong upward trend in the use of nitrous oxide in the 2010s [4, 5, 6], although latest data suggests use may have stabilised or be gradually declining [5, 7]. The increases observed in use may have related to increased access at low cost and in bulk, emergence of 24/7 express delivery services [8], increasing references in popular culture (e.g., [9]) and/or common perceptions of safety [10].

While nitrous oxide has a reputation of being relatively harmless, there are well‐established health risks, particularly associated with heavy and/or frequent use [11, 12]. Most chronic harms result from Vitamin B12 inactivation and are neurological, haematological or psychiatric in nature [10, 11, 12]. Key neurological concerns include myelopathy, polyneuropathy and subacute combined degeneration of the spinal cord [13] and without early intervention, there is a risk of permanent disability [11]. In recent years, there have been increasing reports of significant neurological harm associated with heavy and/or prolonged use [11, 14, 15]. Internationally, and more recently in Australia [16], these poisonings have been linked to larger cylinder/tank options (e.g., 580–2048 g) resulting in prolonged exposure and associated toxicity [1, 17].

In response to rising harm, multiple countries are considering or have implemented, regulatory action to restrict non‐therapeutic access (e.g., [18, 19]), while protecting legitimate use in the hospitality and performance motorsports industries. In Australia, from October 2022, nitrous oxide was reclassified by the Therapeutic Goods Administration as a Schedule 6 poison, prohibiting sales to young people aged under 16 years and mandating product warning labels [20]. Moreover, some state governments have implemented, or are initiating, additional regulatory controls, such as restricted trading hours in South Australia [21] and restricting sales to registered food and beverage businesses in Western Australia [22]. However, supply restrictions must be approached with caution given the risk of stockpiling or substitution with unregulated bulbs and/or higher toxicity inhalants [1, 23].

Responses to rising harm should also include demand and harm reduction measures. However, little is known about the drivers of harmful use in Australia [15], including how commonly and where larger cylinders are accessed, to inform such endeavours. The extent to which consumers are aware of risks associated with heavy and/or prolonged use and are engaging in harm reduction strategies, is also unclear. Further, despite growing evidence relating to severe cases of neurotoxicity [11, 14, 15], little is known about the experience of more minor harms and associated help‐seeking. That is, while there are numerous studies of nitrous oxide in clinical settings (e.g., hospital case studies, poisons registries [3, 24, 25, 26, 27, 28, 29]), these are biased towards more serious harms. To date, the Global Drugs Survey is the only known study outside of clinical settings to investigate harm among people who use nitrous oxide [29].

Given these gaps, this paper investigates the following among cross‐sectional sentinel samples of people who regularly use ecstasy and/or other illicit stimulants in Australia (2021–2023):

- how nitrous oxide is being sourced and products/forms being used;
- factors associated with heavier patterns of use, with a particular focus on modes of acquisition (e.g., 24/7 delivery services);
- awareness of health risks and engagement in harm reduction strategies; and
- self‐reported experience of early neurological symptoms, Vitamin B12 deficiency and associated treatment.

## 2 Methods

The Ecstasy and Related Drug Reporting System (EDRS) is an Australian drug monitoring program that includes annual interviews with non‐representative and cross‐sectional sentinel samples of people recruited from all Australian capital cities. From 2021 to 2023, eligibility criteria comprised: aged ≥ 18 years; used ecstasy and/or other illicit stimulants ≥ 6 times during the preceding 6 months; and resided in the capital city of recruitment for 10 of the preceding 12 months. Participants were recruited through a purposive sampling strategy, with most recruited via Facebook advertising and snowballing (i.e., word of mouth). Following informed consent, participants completed a 60‐minute interview face‐to‐face or via telephone and received AUD40 reimbursement. Full details can be found elsewhere [30]. Ethical approval was granted by the UNSW Human Research Ethics Committee (HC15015) and jurisdictional ethics committees.

### 2.1 Measures

All information collected from participants was self‐reported (see Tables S1–S3 for relevant items from the 2021 to 2023 surveys). Participants were asked whether they had used any nitrous oxide in the past 6 months. Participants who reported past 6‐month use of nitrous oxide were asked a series of follow‐up questions, although there were variations in which questions were asked across the years.

#### 2.1.1 Nitrous Oxide Source and Products/Forms Being Used (Aim 1)

In 2021 and 2023, participants were asked about the size of the products used in the past 6 months (e.g., small 8‐g bulbs and/or larger cylinders; not asked in 2022). In 2021 and 2022, they were also asked where they purchased the nitrous oxide products they used in the past 6 months, and in 2023 those who reported the use of larger cylinders were asked where they purchased these products (given new evidence linking harms with larger cannisters [1, 17]).

#### 2.1.2 Factors Associated With Heavier Patterns of Nitrous Oxide Use (Aim 2)

##### 2.1.2.1 Outcome Measures

In each year, participants were asked how many days they had used nitrous oxide in the past 6 months and the typical and maximum amount used per session.

##### 2.1.2.2 Correlates

In 2021 and 2022, participants were asked where they purchased the nitrous oxide products they used in the past 6 months. Potential factors, chosen a priori, which may also influence patterns of nitrous consumption included: age at time of interview, gender identity (people who identified as non‐binary were excluded from regression analyses due to small numbers), sexual identity, state or territory of residence, accommodation type (recoded as living with parents/family or not), current employment status and current student status (including trade and tertiary studies).

Participants were asked how many days they used ecstasy/MDMA in the past 6 months (used as an indicator of broader engagement in ‘party drug use’; recoded into less than weekly or weekly or more frequent). Participants were also asked if they had any mental health problems in the past 6 months (including issues that they had not spoken to a health professional about).

#### 2.1.3 Awareness of Health Risks and Engagement in Harm Reduction Strategies (Aim 3)

In 2021, participants were asked about awareness of health risks associated with heavy and/or prolonged nitrous oxide use and precautions taken in the past 6 months to reduce the risk of harm associated with nitrous oxide use (harm reduction strategies).

#### 2.1.4 Experience of Early Neurological Symptoms, Diagnosed B12 Deficiency and Help‐Seeking (Aim 4)

Participants were asked about: (i) experience of early neurological symptoms (e.g., tingling, difficulty using hands, and loss of balance) in the past 6 months following use of nitrous oxide which persisted for at least 2 weeks and that they had not experienced before they started using nitrous (2021–2023); (ii) Vitamin B12 deficiency (demonstrated with a blood test) in the past 6 months that they did not have before using nitrous oxide or that had become worse since using nitrous (2021–2022; not asked in 2023 as new recommendations suggest more appropriate markers for B12 deficiency, such as methylmalonic acid (MMA) [11]) and (iii) treatment(s) received for recent neurological symptoms (2023).

### 2.2 Data Analyses

Descriptive analyses (Aims 1, 3 and 4) were conducted using SPSS v28. Percentages were calculated for categorical data, means for normally distributed continuous variables, and medians for continuous data with significant positive skew and/or kurtosis.

To address Aim 2, analyses were conducted using Stata 17, which was the preferred program of the author performing the analysis. Since the question about the source of any size nitrous oxide product was not included in the 2023 survey, analyses were performed on combined 2021 and 2022 data, with repeat participants excluded (i.e., 2022 participants who reported taking part in the 2021 survey were excluded). However, repeat participants have not been excluded in analyses for other aims, as the data are reported by year and excluding them would result in an inaccurate year‐by‐year comparison. There were three outcome measures: frequency of use (less than monthly; monthly or more), quantity used in a ‘typical’ session (recoded into 1–9; 10–29; ≥ 30 bulbs) and quantity used in ‘heaviest session’ in the past 6 months (recoded into 1–9; 10–29; ≥ 30 bulbs). Binomial logistic regression was used to determine association of potential factors with frequency of recent use; multinomial logistic regression models were used to determine the association of mode of acquiring nitrous oxide with the quantity of bulbs used per ‘typical’ session and ‘heavy’ session, respectively. All three models included the mode of acquiring nitrous (24/7 delivery service; corner store; friend; other method) and other correlates were identified a priori. Univariable models were run first for each correlate; variables where *p* < 0.05 were included in multivariable models, noting age, gender and jurisdiction were retained in multivariable models regardless of statistical significance. Our approach aimed to balance control of confounders with limiting the number of variables to ensure a parsimonious model. Parsimony is important for maintaining numerical stability and preventing overfitting. We achieved this by including all potential confounders (e.g., age, gender, etc.) a priori in the adjusted models, then other variables that were purposefully selected based on domain knowledge and conceptual relevance followed, followed by the application of a *p* < 0.05 threshold to retain only those contributing meaningfully to the model. This approach has been used elsewhere. We then assessed the numerical stability of the adjusted models using variance inflation factors, which were within acceptable limits. Due to nonlinearity, age was categorised for regression models (18–19; 20–24; 25 years or older).

Adjusted odds ratios (AOR) are reported for binomial regression and adjusted relative risk ratios (ARRR) for multinomial logistic regression, with associated 95% confidence intervals (CI). Model diagnostics showed no evidence of collinearity or influential outliers. Complete case analyses were used.

## 3 Results

### 3.1 Sample Characteristics

Of 774, 700 and 708 EDRS participants in 2021, 2022 and 2023 respectively, 379 (49%), 315 (45%) and 284 (40%) reported recent (past 6 month) nitrous oxide use. Nitrous oxide was used on a median of 4 days in the 6 months preceding the interview in 2021 (interquartile range [IQR] = 2–10), 4 days in 2022 (IQR = 2–10), and 3 days in 2023 (IQR = 2–10).

The median age of these respondents ranged between 22 and 23 years (IQR = 20–28) and between 57% and 68% identified as male. Table S1 presents the demographic statistics according to data collection year.

### 3.2 How Nitrous Oxide Was Sourced and Product Types Used

In 2021 and 2022, the most commonly reported source of any nitrous oxide in the past 6 months was corner/convenience stores (61% and 58%), followed by 24/7 delivery services (18% and 17%) (Table 1). In 2023, participants were asked where they accessed larger cylinders; corner stores remained the most commonly reported source (28%), followed by friends (24%) and then online shops (16%).

In 2021, a very small proportion (6%) reported using larger canisters; this increased to 26% in 2023 (Table 1).

**TABLE 1:** Nitrous oxide source and products/forms used, Ecstasy and Related Drug Reporting System, 2021–2023.

Variable	2021	2022	2023
	% (*n*)	% (*n*)	% (*n*)
Where did you purchase the nitrous oxide you used in the past 6 months?^a^, ^b^	*n =* 379	*n* = 315	*n* = 74
Corner store/convenience store	60.4 (229)	57.5 (181)	28.4 (21)
24/7 delivery service	17.7 (67)	17.1 (54)	10.8 (8)
Did not purchase/given/traded	16.4 (62)	12.7 (40)	16.2 (12)
Friend/acquaintance	10.8 (41)	14.3 (45)	24.3 (18)
Online shop, but not a 24/7 delivery service	6.9 (26)	6.3 (20)	16.2 (12)
Head shop	4.0 (15)	4.1 (13)	—
Hospitality/catering shop	2.9 (11)	1.9 (6)	—
Dealer, but not a 24/7 delivery service	2.6 (10)	—	—
Other	2.4 (9)	5.1 (16)	—
Don't know	0 (0)	—	—
Skip	0 (0)	—	—
Which form/s of nitrous oxide did you use in the past 6 months?^a^	*n =* 379	*/*	*n* = 281
Small bulbs (~8 g)	97.1 (368)		89.3 (251)
Bottle/cylinder (> 8–1000 g)	4.5 (17)		18.9 (53)
Bottle/cylinder/tank (≥ 1000 g)	1.8 (7)		—
Any larger cylinder^c^	6.3 (24)		26.3 (74)
Don't know	—		—

*Note*: /, question was not included in the questionnaire in this reporting period.—*n* ≤ 5 not 0.

^a^ Multiple responses were allowed so the total may exceed 100%.

^b^ In 2023, the survey only asked where larger cylinders (> 8 g bulbs) were purchased in the past 6 months.

^c^ When combining response options for bottles ‘> 8–1000 g’ and ‘≥ 1000 g’.

### 3.3 Factors Associated With Heavier Patterns of Use, 2021–2022 Data

In the first multivariable model (Table 2), acquiring nitrous oxide via corner/convenience stores and acquiring nitrous oxide via 24/7 delivery services (relative to other sources) was associated with greater odds of reporting monthly or more frequent nitrous oxide use, adjusting for other covariates. Additionally, weekly or more frequent use of ecstasy and using ≥ 10 bulbs in ‘typical’ sessions were associated with greater odds of reporting monthly or more frequent nitrous oxide use than reporting less‐than‐monthly use, while older age (≥ 20) and living in Queensland or Western Australia were associated with lower odds (see Table 2 for full statistics including reference groups).

**TABLE 2:** Factors associated with monthly or more frequent nitrous oxide use (cross‐tabulation and binomial logistic regression), Ecstasy and Related Drug Reporting System, 2021–2022.

	Less than monthly (*n* = 423; ref.)	Monthly or more (*n* = 241)		
	% (*n*)	% (*n*)	OR (95% CI; *p*)	AOR (95% CI; *p*)
Age				
18–19 years	51 (73)	49 (70)	Ref.	Ref.
20–24 years	67 (188)	33 (92)	0.51 (0.34–0.77; **0.001**)	0.51 (0.31–0.84; **0.009**)
≥ 25 years	67 (161)	33 (79)	0.51 (0.33–0.78; **0.002**)	0.52 (0.31–0.88; **0.014**)
Jurisdiction				
ACT	51 (50)	49 (48)	1.34 (0.77–2.33; 0.293)	1.10 (0.55–2.18; 0.790)
NSW	58 (63)	42 (45)	Ref.	Ref.
NT	68 (34)	32 (16)	0.62 (0.30–1.27; 0.188)	0.62 (0.27–1.43; 0.260)
Queensland	68 (52)	32 (24)	0.66 (0.36–1.22; 0.186)	0.37 (0.17–0.79; **0.011**)
SA	65 (37)	35 (20)	0.76 (0.39–1.47; 0.411)	0.46 (0.20–1.06; 0.069)
Tasmania	75 (41)	25 (14)	0.48 (0.23–0.98; **0.044**)	0.4920 (0.17–1.12; 0.121)
Victoria	70 (80)	30 (35)	0.61 (0.35–1.06; 0.081)	0.58 (0.28–1.20; 0.144)
WA	63 (67)	37 (40)	0.84 (0.48–1.45; 0.521)	0.41 (0.20–0.81; **0.011**)
Gender identity				
Male	63 (269)	37 (158)	1.07 (0.76–1.51; 0.695)	0.92 (0.60–1.41; 0.697)
Female	65 (135)	35 (74)	Ref.	Ref.
Sexual identity				
Heterosexual	64 (293)	36 (168)	Ref.	/
Other	64 (128)	36 (72)	0.98 (0.69–1.39; 0.913)	/
Currently employed				
Yes	65 (354)	35 (190)	0.74 (0.49–1.11; 0.146)	/
No	58 (69)	42 (50)	Ref.	/
Current student				
Yes	65 (210)	35 (111)	0.87 (0.64–1.20; 0.401)	/
No	62 (213)	38 (129)	Ref.	/
Living with parents				
Yes	60 (119)	40 (81)	1.29 (0.92–1.82; 0.139)	/
No	66 (304)	34 (160)	Ref.	/
Weekly use of any ecstasy				
Yes	44 (37)	56 (48)	2.54 (1.60–4.04; **< 0.001**)	1.97 (1.10–3.53; **0.023**)
No	66 (366)	34 (187)	Ref.	Ref.
Mental health problem				
Yes	61 (242)	39 (157)	1.42 (1.02–1.97; **0.039**)	1.50 (1.00–2.25; **0.052**)
No	69 (177)	31 (81)	Ref.	Ref.
N_2_0 source—delivery service				
Yes	52 (60)	48 (58)	1.90 (1.27–2.84; **0.002**)	2.43 (1.34–4.40; **0.003**)
No	66 (358)	34 (182)	Ref.	Ref.
N_2_0 source—convenience store				
Yes	54 (212)	46 (181)	2.98 (2.10–4.23; **< 0.001**)	3.51 (2.05–6.02; **< 0.001**)
No	78 (206)	22 (59)	Ref.	Ref.
N_2_0 source—friend				
Yes	71 (58)	29 (24)	0.69 (0.42–1.14; 0.149)	/
No	63 (360)	37 (216)	Ref.	/
N_2_0 source—other				
Yes	70 (146)	30 (64)	0.68 (0.48–0.96; **0.029**)	1.47 (0.86–2.52; 0.156)
No	61 (272)	39 (176)	Ref.	Ref.
Typical quantity per session				
1–9 bulbs	77 (285)	23 (83)	Ref.	Ref.
10–29 bulbs	51 (93)	49 (88)	3.25 (2.22–4.75; **< 0.001**)	2.73 (1.76–4.24; **< 0.001**)
≥ 30 bulbs	34 (37)	66 (67)	6.77 (4.19–10.93; **< 0.001**)	6.71 (3.79–11.86; **< 0.001**)

*Note: p* values should be interpreted with caution due to multiple parameters in the model. People who identified as non‐binary were excluded due to small numbers. /, Not included in adjusted model. Bold values indicates statistical significance (*p* < 0.05).

Abbreviations: ACT, Australian Capital Territory; AOR, adjusted odds ratios; CI, confidence interval; NSW, New South Wales; NT, Northern Territory; OR, odds ratio; SA, South Australia; WA, Western Australia.

Similarly, acquiring nitrous oxide via 24/7 delivery services and acquiring nitrous oxide via corner/convenience stores were associated with a greater likelihood of reporting higher quantities of bulbs (i.e., 10–29 and ≥ 30 bulbs) used in both ‘typical’ and ‘heavy sessions’, although there were some slight nuances (Tables 3 and 4, respectively). Specifically, acquiring nitrous via corner/convenience stores was associated with higher likelihood of using 10–29 and ≥ 30 bulbs in a typical session and higher likelihood of using 10–29 bulbs in a heavy session, when compared to those using 1–9 bulbs. Meanwhile, acquiring nitrous via 24/7 delivery services was associated with higher odds of using 10–29 and ≥ 30 bulbs in a typical session and higher odds of using ≥ 30 bulbs in a heavy session, when compared to those using 1–9 bulbs. Additionally, living in Western Australia or South Australia, male gender, weekly or more frequent use of ecstasy and monthly or more frequent use of nitrous oxide were associated with a greater likelihood of reporting higher quantities of bulbs used in both ‘typical’ and ‘heavy sessions’ (Tables 3 and 4, respectively). However, *p* values should be interpreted with caution due to multiple parameters in the model.

**TABLE 3:** Factors associated with higher quantity use in ‘typical’ sessions (cross‐tabulation and multi‐nomial logistic regression), Ecstasy and Related Drug Reporting System, 2021–2022.

	1–9 bulbs (*n* = 369; ref.)	10–29 bulbs (*n* = 181)			≥ 30 bulbs (*n* = 101)		
	% (*n*)	% (*n*)	RRR (95% CI; *p*)	ARRR (95% CI; *p*)	% (*n*)	RRR (95% CI; *p*)	ARRR (95% CI; *p*)
Age							
18–19 years	52 (75)	32 (46)	Ref.	Ref.	15 (22)	Ref.	Ref.
20–24 years	62 (169)	23 (63)	0.61 (0.38–0.97; 0.037)	0.97 (0.57–1.65; 0.905)	15 (40)	0.81 (0.45–1.45; 0.474)	1.54 (0.75–3.15; 0.238)
≥ 25 years	53 (125)	30 (71)	0.93 (0.58–1.48; 0.748)	1.43 (0.81–2.52; 0.219)	17 (39)	1.06 (0.59–1.93; 0.839)	2.01 (0.94–4.33; 0.073)
Jurisdiction							
ACT	60 (58)	26 (25)	1.06 (0.55–2.04; 0.859)	1.20 (0.55–2.65; 0.644)	14 (13)	1.20 (0.51–2.83; 0.685)	1.54 (0.53–4.45; 0.423)
NSW	63 (64)	25 (26)	Ref.	Ref.	13 (12)	Ref.	Ref.
NT	58 (28)	33 (16)	1.41 (0.65–3.02; 0.382)	2.03 (0.83–4.92; 0.119)	8 (4)	0.76 (0.23–2.57; 0.661)	1.05 (0.27–4.12; 0.939)
Queensland	62 (46)	28 (21)	1.12 (0.56–2.24; 0.740)	1.54 (0.70–3.41; 0.287)	9 (7)	0.81 (0.30–2.22; 0.684)	0.90 (0.27–3.01; 0.862)
SA	47 (26)	33 (18)	1.70 (0.80–3.62; 0.166)	4.04 (1.63–10.02; 0.003)	20 (11)	2.26 (0.88–5.76; 0.089)	5.35 (1.65–17.33; 0.005)
Tasmania	72 (39)	19 (10)	0.63 (0.27–1.45; 0.278)	1.13 (0.43–2.97; 0.804)	9 (5)	0.68 (0.22–2.09; 0.505)	0.83 (0.21–3.31; 0.795)
Victoria	58 (67)	25 (29)	1.07 (0.57–2.00; 0.844)	1.49 (0.69–3.25; 0.311)	17 (19)	1.51 (0.68–3.37; 0.311)	1.29 (0.45–3.72; 0.635)
WA	38 (41)	34 (36)	2.16 (1.14–4.09; 0.018)	3.86 (1.83–8.13; **< 0.001**)	28 (30)	3.90 (1.80–8.48; **0.001**)	9.40 (3.58–24.72; **< 0.001**)
Gender identity							
Male	53 (225)	31 (129)	1.76 (1.17–2.64; **0.006**)	1.99 (1.26–3.15; **0.003**)	16 (68)	1.48 (0.90–2.42; 0.122)	1.89 (1.05–3.42; 0.034)
Female	65 (132)	21 (43)	Ref.	Ref.	13 (27)	Ref.	Ref.
Sexual identity							
Heterosexual	55 (253)	27 (125)	Ref.	/	17 (78)	Ref.	/
Other	60 (116)	28 (54)	0.94 (0.64–1.39; 0.763)	/	11 (22)	0.62 (0.37–1.04; 0.068)	/
Currently employed							
Yes	57 (307)	29 (155)	1.18 (0.72–1.95; 0.505)	1.34 (0.75–2.40; 0.328)	14 (74)	0.54 (0.32–0.92; **0.022**)	0.56 (0.31–1.15; 0.123)
No	54 (61)	23 (26)	Ref.	Ref.	24 (27)	Ref.	Ref.
Current student							
Yes	62 (195)	27 (83)	0.76 (0.53–1.08; 0.124)	0.98 (0.63–1.50; 0.722)	11 (35)	0.48 (0.30–0.76; **0.002**)	0.61 (0.35–1.06; 0.080)
No	52 (174)	29 (98)	Ref.	Ref.	19 (65)	Ref.	Ref.
Living with parents/family							
Yes	52 (103)	29 (56)	1.16 (0.78–1.71; 0.462)	/	18 (36)	1.43 (0.90–2.28; 0.133)	/
No	75 (266)	29 (125)	Ref.	/	14 (65)	Ref.	/
Weekly use of any ecstasy							
Yes	36 (30)	37 (31)	2.27 (1.33–3.89; **0.003**)	1.57 (0.84–2.91; 0.909)	27 (23)	3.29 (1.86–6.18; **< 0.001**)	2.56 (1.25–5.27; **0.010**)
No	59 (323)	27 (147)	Ref.	Ref.	13 (73)	Ref.	Ref.
Mental health problem							
Yes	57 (220)	28 (107)	1.00 (0.69–1.44; 0.999)	/	16 (62)	1.08 (0.69–1.71; 0.732)	/
No	57 (146)	28 (71)	Ref.	/	15 (38)	Ref.	/
N_2_0 source—delivery service							
Yes	39 (45)	35 (41)	2.11 (1.32–3.37; **0.002**)	2.94 (1.64–5.27; **< 0.001**)	26 (30)	3.00 (1.77–5.10; **< 0.001**)	3.84 (1.818.15; **< 0.001**)
No	60 (320)	26 (138)	Ref.	Ref.	13 (71)	Ref.	Ref.
N_2_0 source—convenience store							
Yes	49 (191)	34 (131)	2.49 (1.68–3.67; **< 0.001**)	1.92 (1.19–3.08; **0.007**)	17 (64)	1.58 (1.00–2.48; 0.050)	0.88 (0.48–1.60; 0.675)
No	67 (174)	19 (48)	Ref.	Ref.	14 (37)	Ref.	Ref.
N_2_0 source—friend							
Yes	66 (53)	20 (16)	0.58 (0.32–1.04; 0.069)	/	14 (11)	0.72 (0.36–1.44; 0.350)	/
No	55 (312)	29 (163)	Ref.	/	16 (90)	Ref.	/
N_2_0 source—other							
Yes	60 (123)	22 (46)	0.68 (0.46–1.01; 0.059)	/	18 (36)	1.09 (0.69–1.73; 0.715)	/
No	55 (242)	30 (133)	Ref.	Ref.	15 (65)	Ref.	Ref.
Frequency of use							
Less than monthly	69 (285)	23 (93)	Ref.	Ref.	8 (34)	Ref.	Ref.
Monthly or more	35 (83)	37 (88)	3.25 (2.22–4.75; **< 0.001**)	2.95 (1.89–4.60; **< 0.001**)	28 (67)	6.77 (4.19–10.93; **< 0.001**)	6.82 (3.81–12.19; **< 0.001**)

*Note: p* values should be interpreted with caution due to multiple parameters in the model. People who identified as non‐binary were excluded due to small numbers. /, Not included in adjusted model. Bold values indicates statistical significance (*p* < 0.05).

Abbreviations: ACT, Australian Capital Territory; ARRR, adjusted relative risk ratios; CI, confidence interval; NSW, New South Wales; NT, Northern Territory; RRR, relative risk ratios; SA, South Australia; WA, Western Australia.

**TABLE 4:** Factors associated with higher quantity use in ‘heavy’ sessions (cross‐tabulation and multi‐nomial logistic regression), Ecstasy and Related Drug Reporting System, 2021–2022.

	1–9 bulbs (*n* = 265; ref)	10–29 bulbs (*n* = 201)			≥ 30 bulbs (*n* = 185)		
	% (*n*)	% (*n*)	RRR (95% CI; *p*)	ARRR (95% CI; *p*)	% (*n*)	RRR (95% CI; *p*)	ARRR (95% CI; *p*)
Age							
18–19 years	35 (50)	35 (50)	Ref.	Ref.	30 (43)	Ref.	Ref.
20–24 years	44 (120)	28 (76)	0.63 (0.39–1.03; 0.065)	1.14 (0.65–2.03; 0.644)	28 (76)	0.74 (0.45–1.21; 0.230)	1.66 (0.86–3.19; 0.129)
≥ 25 years	40 (95)	31 (74)	0.78 (0.47–1.28; 0.324)	1.30 (0.69–2.32; 0.452)	28 (66)	0.81 (0.48–1.35; 0.416)	1.74 (0.87–3.51; 0.119)
Jurisdiction							
ACT	46 (44)	32 (31)	0.91 (0.48–1.73; 0.778)	0.76 (0.34–1.67; 0.494)	22 (21)	0.87 (0.43–1.80; 0.716)	1.10 (0.43–2.86; 0.838)
NSW	43 (44)	33 (34)	Ref.	Ref.	24 (24)	Ref.	Ref.
NT	42 (20)	40 (19)	1.23 (0.57–2.66; 0.600)	1.85 (0.75–4.59; 0.182)	19 (9)	0.83 (0.33–2.09; 0.685)	1.72 (0.54–5.45; 0.358)
Queensland	47 (35)	28 (21)	0.78 (0.38–1.57; 0.480)	1.06 (0.47–2.40; 0.893)	24 (18)	0.94 (0.44–2.01; 0.879)	1.59 (0.60–4.23; 0.355)
SA	31 (17)	36 (20)	1.52 (0.69–3.34; 0.295)	3.93 (1.12–7.66; 0.028)	33 (18)	1.94 (0.85–4.45; 0.117)	6.01 (1.97–18.33; **0.002**)
Tasmania	54 (29)	26 (14)	0.62 (0.29–1.36; 0.237)	1.35 (0.55–3.30; 0.515)	20 (11)	0.70 (0.30–1.63; 0.404)	1.78 (0.58–5.52; 0.316)
Victoria	43 (50)	26 (30)	0.78 (0.41–1.47; 0.436)	1.23 (0.56–2.69; 0.613)	30 (35)	1.28 (0.66–2.48; 0.458)	2.28 (0.90–5.77; 0.082)
WA	24 (26)	30 (32)	1.59 (0.80–3.16; 0.182)	2.65 (1.20–5.86; **0.016**)	46 (49)	3.46 (1.74–6.88; **< 0.001**)	10.89 (4.39–27.00; **< 0.001**)
Gender identity							
Male	37 (156)	32 (135)	1.52 (1.02–2.26; **0.040**)	1.57 (0.99–2.50; 0.057)	31 (131)	1.87 (1.22–2.84; **0.004**)	2.41 (1.39–4.16; **0.002**)
Female	50 (100)	28 (57)	Ref.	Ref.	22 (45)	Ref.	Ref.
Sexual identity							
Heterosexual	40 (184)	29 (134)	Ref.	/	31 (138)	Ref.	/
Other	42 (81)	34 (65)	1.10 (0.74–1.64; 0.630)	/	24 (46)	0.76 (0.50–1.16; 0.198)	/
Currently employed							
Yes	42 (223)	31 (164)	0.86 (0.53–1.40; 0.539)	/	28 (149)	0.78 (0.48–1.27; 0.320)	/
No	37 (42)	32 (36)	Ref.	/	32 (36)	Ref.	/
Current student							
Yes	44 (138)	32 (99)	0.89 (0.62–1.29; 0.546)	1.00 (0.63–1.57; 0.990)	24 (76)	0.65 (0.44–0.95; **0.025**)	0.80 (0.48–1.33; 0.386)
No	38 (127)	30 (102)	Ref.	Ref.	32 (108)	Ref.	Ref.
Living with parents/family							
Yes	37 (72)	30 (59)	1.11 (0.74–1.67; 0.604)	/	33 (64)	1.42 (0.94–2.13; 0.092)	/
No	42 (193)	31 (142)	Ref.	/	27 (121)	Ref.	/
Weekly use of any ecstasy							
Yes	25 (21)	25 (21)	1.38 (0.73–2.61; 0.319)	0.88 (0.41–1.86; 0.733)	50 (42)	3.41 (1.94–5.99; **< 0.001**)	2.53 (1.22–5.25; **0.013**)
No	43 (235)	31 (170)	Ref.	Ref.	25 (138)	Ref.	Ref.
Mental health problem							
Yes	40 (156)	30 (115)	0.92 (0.63–1.34; 0.659)	/	30 (118)	1.25 (0.85–1.85; 0.259)	/
No	42 (106)	33 (85)	Ref.	Ref.	25 (64)	Ref.	Ref.
N_2_0 source—delivery service							
Yes	27 (31)	27 (31)	1.36 (0.80–2.33; 0.260)	1.95 (0.97–3.92; 0.060)	47 (54)	3.08 (1.89–5.04; **< 0.001**)	4.39 (2.10–9.18; **< 0.001**)
No	43 (230)	32 (169)	Ref.	Ref.	25 (130)	Ref.	Ref.
N_2_0 source—convenience store							
Yes	31 (118)	36 (140)	2.83 (1.92–4.17; **< 0.001**)	2.57 (1.42–4.68; **0.002**)	33 (128)	2.77 (1.86–4.12; **< 0.001**)	2.43 (1.25–4.70; **0.009**)
No	55 (143)	23 (60)	Ref.	Ref.	22 (56)	Ref.	Ref.
N_2_0 source—friend							
Yes	44 (35)	31 (25)	0.92 (0.53–1.60; 0.774)	/	25 (20)	0.79 (0.44–1.41; 0.423)	/
No	40 (226)	31 (175)	Ref.	/	29 (164)	Ref.	/
N_2_0 source—other							
Yes	48 (99)	26 (53)	0.59 (0.39–0.88; **0.010**)	1.29 (0.71–2.36; 0.405)	26 (53)	0.66 (0.44–0.99; **0.046**)	1.41 (0.72–2.76; 0.316)
No	37 (162)	33 (147)	Ref.	Ref.	30 (131)	Ref.	Ref.
Frequency of use							
Less than monthly	57 (235)	28 (115)	Ref.	Ref.	15 (62)	Ref.	Ref.
Monthly or more	12 (29)	36 (86)	6.06 (3.76–9.76; **< 0.001**)	6.18 (3.63–10.53; **< 0.001**)	28 (123)	16.08 (9.83–26.29; **< 0.001**)	15.81 (8.82–28.33; **< 0.001**)

*Note: p* values should be interpreted with caution due to multiple parameters in the model. People who identified as non‐binary were excluded due to small numbers. /, Not included in adjusted model. Bold values indicates statistical significance (*p* < 0.05).

Abbreviations: ACT, Australian Capital Territory; ARRR, adjusted relative risk ratios; CI, confidence interval; NSW, New South Wales; NT, Northern Territory; RRR, relative risk ratios; SA, South Australia; WA, Western Australia.

### 3.4 Awareness of Health Risks and Engagement in Harm Reduction Strategies, 2021

While most participants could identify at least one neurological health risk associated with nitrous oxide use (e.g., brain damage 63%; nerve damage 20%), only one‐fifth (19%) identified the risk of Vitamin B12 depletion (the etiological cause of neurological damage) and 17% were unaware of any risks (no 15%; don't know 2%) (Table 5). The most commonly reported precautions to reduce risk were limiting frequency of use (36%) and quantity used per session (30%), while one‐tenth (9%) reported Vitamin B12 supplements or dietary changes. Approximately one‐third (36%) reported taking no precautions.

**TABLE 5:** Awareness of risks and engagement in harm reduction strategies, Ecstasy and Related Drug Reporting System, 2021.

	% (*n*)
Do you know of any health risks associated with heavy and/or prolonged nitrous oxide use?^a^	*n* = 379
Reported any health risk	82.8 (314)
Brain damage	62.7 (237)
Nerve damage/peripheral neuropathy^b^	20.0 (75)
Vitamin B12 depletion	19.0 (72)
Memory problems	13.5 (51)
Numbness/tingling	9.3 (35)
Difficulty walking or balancing	7.7 (29)
Difficulty using hands and fingers	5.0 (19)
Psychosis	4.5 (17)
Fatigue/weakness	4.0 (15)
Other mental health problems	2.9 (11)
Weakened immune system	2.4 (9)
Other	12.2 (46)
No	14.8 (56)
Don't know	2.4 (9)
In the past 6 months, did you take any precautions to reduce risks?	*n =* 379
Limited frequency of use	36.4 (138)
Limited the quantity per session	30.3 (115)
Sat down while using	15.6 (59)
Took regular breaths of oxygen	7.9 (30)
Used a vitamin B12 supplement^c^	7.9 (30)
Did not use alone	7.7 (29)
Limited amount of rebreathing (e.g., from the balloon)	5.3 (20)
Did not rebreathe (e.g., from the balloon)	4.5 (17)
Only used food or medical grade nitrous oxide	3.7 (14)
Used in a well‐ventilated area (not small and enclosed)	3.4 (13)
Included vitamin B12 in diet^c^	2.9 (11)
Filtered the nitrous oxide	2.6 (10)
Protected hands (e.g., with gloves) when using nang cracker	2.1 (8)
Other (specify)	6.3 (24)
No	35.6 (135)

^a^ Multiple responses were allowed so the total may exceed 100%.

^b^ Other responses entered mentioning paralysis or spinal damage were merged into this category.

^c^ A total of 9% (*n* = 35) reported either B12 supplementation or adding B12 to their diet.

### 3.5 Experience of Persistent Neurological Symptoms Only Experienced Post‐Use

The percentage of participants who had recently used nitrous oxide and reported experiencing persistent neurological symptoms post‐use in the past 6 months rose from 5% in 2021 to 14% in 2023 (Table 6). In 2023, few participants (*n* ≤ 5) reported receiving treatment for these symptoms. Few participants (*n* ≤ 5) reported experiencing a Vitamin B12 deficiency (diagnosed with a blood test) post‐use in 2021 (albeit with 6% unsure), while 3% reported a B12 deficiency in 2022.

**TABLE 6:** Experience of persistent neurological symptoms post nitrous oxide use, Ecstasy and Related Drug Reporting System, 2021–2023.

	2021	2022	2023
	% (*n*)	% (*n*)	% (*n*)
In the past 6 months, have you experienced the following symptoms?^a^	*n =* 379	*n* = 315	*n* = 283
None of the below	92.9 (353)	89.2 (281)	85.2 (241)
Any neurological symptom^b^	5.3 (20)	9.5 (30)	13.9 (39)
Numbness/tingling around your face or mouth	2.1 (8)	4.1 (13)	5.3 (15)
Numbness/tingling in your hands or feet	2.1 (8)	6.7 (21)	10.6 (30)
Difficulty walking or with your balance	2.1 (8)	4.1 (13)	6.0 (17)
Difficulty with using your hands and fingers	1.6 (6)	4.8 (15)	4.6 (13)
Don't know	—	—	—
Did you receive treatment for these symptoms?	*/*	*/*	*n* = 39
No			97.4 (38)
Yes			—
In the past 6 months, have you experienced a Vitamin B12 deficiency?	*n =* 379	*n* = 311	*/*
No	92.6 (351)	86.5 (269)	
Yes	—	3.2 (10)	
Don't know	6.1 (23)	10.3 (32)	

*Note*: See Tables S2–S4 for full question wording. ‘Skip’ responses were excluded from this table. –*n* ≤ 5 not 0.

^a^ Multiple responses were allowed so the total may exceed 100%.

^b^ Computed if selected ‘yes’ to any of the four neurological symptom responses.

## 4 Discussion

This paper investigated potential drivers of nitrous oxide‐related harms (e.g., market changes and lack of awareness of health risks) and experience of early neurological symptoms (e.g., persistent tingling) to help inform regulatory and other health‐related responses in Australia.

### 4.1 Market Changes

Our findings indicate a shift from almost exclusively sourcing/using small 8 g bulbs to sourcing/using larger cylinders (ranging from about 580 to 2000 g). That is, while the majority of participants who had used nitrous oxide reported the use of small bulbs (~8 g) in both 2021 and 2023, reported use of larger cylinders increased fourfold, from 6% in 2021 to 26% in 2023. This finding is unsurprising given that larger cylinders have flooded the Australian market in recent years and are commonly promoted in multi‐buy specials by convenience store and 24/7 delivery service retailers [31, 32]. However, this shift is of significant concern given the capacity for greater exposure and associated neurotoxicity and links with harm internationally [1, 17] and in Australia [16]. Specifically, this shift should inform harm reduction and regulatory responses and aligns with Western Australia's decision to specify a ban on larger canisters [22]. However, regulators should also consider the possibility that market disruptions and announcements of new regulations restricting sales could drive individuals to stockpile larger cylinders and potentially increase use in the interim. Further, regulations restricting access to nitrous oxide may redirect use towards more harmful alternatives, such as unregulated products or more dangerous inhalants [33, 34], highlighting the need for balanced responses.

The findings also suggest that certain types of retailers may be contributing to more harmful patterns of use. When controlling for other variables, both corner stores and 24/7 delivery services were associated with higher frequency and higher quantity use. However, convenience stores had a larger effect size for higher frequency use, while 24/7 delivery services had a larger effect size for higher quantity use. While we cannot conclude causation based on cross‐sectional data, the latter finding may relate to 24/7 delivery services offering convenient and rapid replenishment of nitrous oxide, leading to higher volume use per session and supports concerns that these newer mobile retailers are likely exacerbating harmful patterns of use [31]. Meanwhile, convenience stores may contribute to higher frequency use due to high visibility displays and window advertising [35], as well as the convenience of 24/7 operating hours in nightlife areas, which may encourage opportunistic purchases. It is also important to acknowledge that the most commonly reported source of nitrous oxide in this sample was convenience/corner stores, highlighting the prominent role these retailers play in the market. Overall, the results raise questions about retailer awareness of potential harm and underscore the importance of new regulations initiated by some state/territory government agencies, including retailer consultation and education [33], Western Australia's restriction of sales to small bulbs by registered food and beverage businesses only [22] and South Australia's limitation on hours of sale, prohibiting 24/7 delivery services [21]. However, new jurisdictional regulations to combat harmful supply patterns are unlikely to be heeded by all retailers given some continue to sell other banned products like vaping devices [33, 36] and many were arguably already operating outside the law by knowingly selling nitrous oxide for the purpose of intoxication. Thus, ongoing market surveillance and consistent enforcement of the law are necessary to deter unscrupulous retailers.

### 4.2 Consumer Awareness of Risk and Engagement in Harm Reduction

Consistent with international studies [10], the findings also highlight the need for greater consumer understanding of the potential health risks associated with nitrous oxide. While most respondents indicated some awareness of neurological risks (e.g., brain damage 63%; nerve damage 20%), only one‐fifth identified Vitamin B12 depletion (the etiological cause of nitrous oxide‐related neurological damage [11, 12]) as a risk. Further, almost one‐fifth could not identify any risks. There was also limited engagement in protective behaviours, although this may reflect generally low‐risk patterns of use among this sample (e.g., low median frequency of use and moderate median amounts used per session). While reports of Vitamin B12 supplementation are encouraging, evidence suggests such efforts have low efficacy without first ceasing use [37]. Thus, more comprehensive education, encompassing not only symptoms indicative of harm, but also mechanisms of harm, may be advisable.

### 4.3 Experience of Neurological Symptoms

Participant reports of persistent neurological symptoms post‐use have also increased in EDRS samples. Specifically, while few respondents (5%) reported any neurological symptom in 2021—comparable to 3% identified in the 2014–2016 Global Drugs Survey [29]—this rose to 14% in 2023. While not clinically verified, these survey findings suggest that there may be a small but increasing number of individuals using nitrous oxide in harmful ways and/or more vulnerable people using, such as females of reproductive age and vegans/vegetarians with higher incidence of Vitamin B12 deficiency [29]. While further statistical investigations are precluded in this study due to sample sizes, future research would benefit from investigating light to heavy use and the spectrum and severity of associated symptoms/harm (i.e., dose effects). The available case series ([27, 28, 37]) and media reports [16] suggest those experiencing severe harm and requiring lengthy hospital stays report using hundreds of bulbs or multiple larger cylinders daily for weeks or months. A greater understanding of the trajectory and progression to this level of use, along with the associated risk factors and severity of dependence, is essential for informing effective prevention and harm reduction efforts. Overall, the findings suggest a broader spectrum of nerve damage (i.e., including early signs of damage before severe disability) may be occurring and further underscores the need to raise capacity for early recognition and response to avoid escalation of harm and long‐term damage. Targeted education in settings beyond the point of treatment, such as music festivals which are popular events for use [38], should be considered for prevention and early intervention [15].

### 4.4 Treatment for Neurological Symptoms

Few participants (*n* < 5) experiencing persistent neurological symptoms reported receiving treatment. While the reasons for not receiving treatment are unknown, this finding again highlights the importance of targeted public health campaigns aimed at raising awareness of early neurological and psychiatric symptoms associated with nitrous oxide use (e.g., tingling) and the need to seek medical help early (when experiencing these symptoms) to reduce the risk of permanent harm. It also highlights the importance of education for clinicians in both primary and tertiary healthcare settings on the psychiatric and neurological symptoms associated with use, along with appropriate screening and treatment regimes. Finally, guidance should be provided to consumers and clinicians on initiating conversations about nitrous oxide use in healthcare settings to encourage more honest dialogue and better health outcomes, given young people may face barriers of shame and/or fear of legal issues [1, 18, 38].

### 4.5 Limitations

Several limitations should be considered. First, due to sampling methods, the findings cannot be seen as representative of the wider population of people who use nitrous oxide (e.g., those living in regional or rural areas, individuals under 18 years old or those who do not use stimulants). Second, Therapeutic Goods Administration‐mandated product warnings have come into effect since data collection in 2021 [20], which may have increased awareness of the risk of nerve damage and encouraged greater engagement in risk mitigation strategies. Consequently, the impacts of regulatory changes on awareness of harms and harm reduction behaviours should be investigated in future surveys. Third, although self‐reports of neurological symptoms increased across years, they remained relatively rare—it would therefore be useful to repeat the research with a larger sample to enable investigation of dose effects and correlates of harm. Fourth, while research suggests self‐report methods among people who use drugs are sufficiently reliable and valid [39], we cannot verify responses—future research would therefore benefit from more accurate measurement of neurological harms (e.g., a clinical assessment). Finally, benefits of use were not explored, which is important for informing contextually relevant interventions [15].

## 5 Conclusions

Findings from this study have implications for ongoing surveillance of nitrous oxide use, harms and market trends in Australia, particularly given the shifting regulatory/risk environment and indications of increasing neurological harms associated with larger canister use [16]. The findings also reinforced the need for evidence‐based interventions targeting retailers, consumers and clinicians. Finally, interventions beyond the point of treatment should be urgently considered.

## Author Contributions

J.G., S.L. and J.S. conceived the study. All authors had input on the study design. J.G. and C.K. prepared and analysed the data. J.G. drafted the manuscript. All authors had input on revising the manuscript critically for important intellectual content. All authors approved the final version to be published. Each author certifies that their contribution to this work meets the standards of the International Committee of Medical Journal Editors.

## Conflicts of Interest

R.S. has received untied educational funds from Seqirus. A.P. has received untied educational grants from Seqirus and Mundipharma for study of opioid medications. Simon Lenton has served as an unpaid member of an Advisory Board for Mundipharma. Funding from this organisation has now ceased and these organisations had no role in study design, conduct or reporting. There are no other relevant interests to declare.

## Supporting information


**Data S1:** Supporting Information.

## Data Availability

The data that support the findings of this study are available on request from the corresponding author. The data are not publicly available due to privacy or ethical restrictions.
