# Supplementary material for: Nitrous Oxide Sourcing, Use and Harms: Insights From Australians Who Use Ecstasy/MDMA and Other Illicit Stimulants
Source: Drug Alcohol Rev. 2025 Sep 3;44(7):2113–26. doi: 10.1111/dar.70032 (PMC12581930; doi:10.1111/dar.70032)
Supplement: Supplementary file 1 — Data S1: Supporting Information. [file DAR-44-2113-s001.docx]

# Supplementary tables

***Table S1. Demographics characteristics of participants who reported past 6-month use of nitrous oxide, Ecstasy and Related Drug Reporting System, nationally, 2021-2023***

|  | 2021  N=379 | 2022  N=315 | 2023  N=284 |
| --- | --- | --- | --- |
| Median age (years; IQR) | 23 (20-26) | 22 (20-26) | 22 (20-28) |
| Gender identity  Male  Female  Non-binary  Other | 67.5 (256)  29.8 (113)  2.4 (9)  - | 58.9 (185)  35.0 (110)  5.1 (16)  - | 57.2 (162)  39.9 (113)  2.5 (7)  - |
| Jurisdiction  New South Wales  Australian Capital Territory  Victoria  Tasmania  South Australia  Western Australia  Northern Territory  Queensland | 17.9 (68)  14.5 (55)  15.8 (60)  11.1 (42)  8.7 (33)  11.9 (45)  11.3 (43)  8.7 (33) | 13.0 (41)  15.2 (48)  19.4 (61)  6.0 (19)  8.9 (28)  22.2 (70)  -  13.7 (43) | 16.5 (47)  18.7 (53)  14.8 (42)  9.2 (26)  7.4 (21)  17.6 (50)  4.6 (13)  11.3 (32) |
| Sexual identity  Straight or heterosexual  Bisexual  Queer  Lesbian, gay or homosexual  Different identity | 69.1 (262)  16.9 (64)  7.4 (28)  4.5 (17)  2.1 (8) | 69.2 (216)  18.6 (58)  5.4 (17)  4.5 (14)  2.2 (7) | 70.0 (198)  18.0 (51)  3.9 (11)  6.7 (19)  - |
| Median years of school education (IQR) | 12 (12-12) | 12 (12-12) | 12 (12-12) |
| % Post-school qualification(s)  No  Yes | 45.4 (172)  54.6 (207) | 46.7 (147)  53.3 (168) | 43.3 (123)  56.7 (161) |
| % Currently studying  No  Yes | 49.6 (188)  50.4 (191) | 53.0 (167)  47.0 (148) | 56.0 (159)  44.0 (125) |
| % Current employment status  Not employed  Full time  Part time/casual  Self employed  Other | 19.5 (74)  24.8 (94)  50.9 (193)  4.7 (46)  0 (0) | 15.9 (50)  28.9 (91)  47.6 (150)  7.3 (23)  - | 14.8 (42)  35.2 (100)  46.5 (132)  3.5 (10)  0 (0) |
| Current median weekly income $ (IQR) (n) | 600 (375-961) (366) | 700 (480-1153) (300) | 750 (495-1255) (274) |
| % Current accommodation  Rented house/flat  Parents/family house  Boarding house/hostel  Own house/flat  Other | 57.0 (216)  30.9 (117)  5.3 (20)  3.7 (14)  3.2 (12) | 59.0 (186)  28.6 (90)  1.9 (6)  6.7 (21)  3.8 (12) | 58.5 (166)  31.0 (88)  -  5.3 (15)  3.5 (10) |

- n≤5 not 0. IQR, interquartile range.

***Table S2. Ecstasy and Related Drug Reporting System nitrous oxide module, 2021***

| # | Skip | Question | Response option |
| --- | --- | --- | --- |
|  |  | The next few questions are about your use of nitrous oxide, how you have accessed nitrous, and any harms that you may be aware of and/or experienced in relation to use of nitrous oxide. | descriptive |
| 1 |  | Where did you purchase the nitrous oxide you used in the past 6 months?  Head shops sell cannabis paraphernalia and items related to countercultures.  (Multiple responses allowed) | Checkbox, required   \| 0 \| Did not purchase/given/traded \| \| --- \| --- \| \| 1 \| Friend/acquaintance \| \| 2 \| Dealer (not a 24/7 delivery service) \| \| 3 \| 24/7 delivery service \| \| 4 \| Corner store/convenience store \| \| 5 \| Head shops (e.g., Off Ya Tree) \| \| 6 \| Adult stores \| \| 7 \| Online shop (but not a 24/7 delivery service) \| \| 8 \| Hospitality/catering/kitchen shop \| \| 9 \| Other (specify) \| \| 10 \| Don’t know \| \| 11 \| Skip question \| |
| 2 | Ask only if Q1 = 9 | Specify other source |  |
| 3 | Skip if Q1 only=0 or if only provide one response in Q1 | Where did you most commonly purchase nitrous oxide in the past 6 months? | Radio, required   \| 1 \| Friend/acquaintance \| \| --- \| --- \| \| 2 \| Dealer (not a 24/7 delivery service) \| \| 3 \| 24/7 express delivery service \| \| 4 \| Corner store/convenience store \| \| 5 \| Head shops (e.g. Off Ya Tree) \| \| 6 \| Adult stores \| \| 7 \| Online shop (but not a 24/7 delivery service) \| \| 8 \| Hospitality/catering/kitchen shop \| \| 9 \| Other (specify) \| \| 10 \| Don’t know \| \| 11 \| Skip question \| |
| 4 | Ask only if Q3 = 9 | Specify other source |  |
| 5 |  | Which form/s of nitrous oxide did you use in the past 6 months?  (Multiple responses allowed)  SHOW FLASHCARD | Checkbox, required   \| 1 \| Small bulbs (~8g) \| \| --- \| --- \| \| 2 \| Bottle/cylinder/tank (≤1000g) \| \| 3 \| Bottle/cylinder/tank (>1000g) \| \| 4 \| Don’t know \| \| 5 \| Skip question \| |
| 6 |  | How did you inhale nitrous oxide in the past 6 months?  (Multiple responses allowed)  DO NOT READ OUT RESPONSES | Checkbox, required   \| 1 \| From a balloon \| \| --- \| --- \| \| 2 \| Directly from a whipped cream dispenser \| \| 3 \| From a bag (not over your head) \| \| 4 \| From a bag (over your head) \| \| 5 \| From a mask \| \| 6 \| Other (specify) \| \| 7 \| Don’t know \| \| 8 \| Skip question \| |
| 7 | Ask only if Q6 = 6 | Specify other inhalation method |  |
| 10 |  | Do you know of any health risks associated with heavy and/or prolonged nitrous oxide use?  (Multiple responses allowed)  DO NOT READ OUT RESPONSES | Checkbox, required   \| 0 \| No \| \| --- \| --- \| \| 1 \| Vitamin B12 depletion \| \| 2 \| Numbness/tingling \| \| 3 \| Difficulty walking or balancing \| \| 4 \| Difficulty using hands and fingers \| \| 5 \| Nerve damage/peripheral neuropathy \| \| 6 \| Memory problems \| \| 7 \| Fatigue/weakness \| \| 8 \| Weakened immune system \| \| 9 \| Psychosis \| \| 10 \| Other mental health problems \| \| 11 \| Brain damage from reduced oxygen \| \| 12 \| Fertility problems \| \| 13 \| Other (specify) \| \| 14 \| Don’t know \| \| 15 \| Skip question \| |
| 11 | Ask only if Q10 = 12 | Specify other health risk |  |
| 12 |  | In the past 6 months, did you take any precautions to reduce risks associated with nitrous oxide use?  (Multiple responses allowed)  DO NOT READ OUT RESPONSES | Checkbox, required   \| 0 \| No \| \| --- \| --- \| \| 1 \| Sat down while using \| \| 2 \| Limited the quantity per session \| \| 3 \| Limited frequency of use \| \| 4 \| Used in a well-ventilated area (not small and enclosed) \| \| 5 \| Did not use alone \| \| 6 \| Did not not rebreathe (e.g. from the balloon) \| \| 7 \| Limited amount of rebreathing (e.g. from the balloon) \| \| 8 \| Took regular breaths of oxygen \| \| 9 \| Filtered the nitrous oxide \| \| 10 \| Used a vitamin B12 supplement \| \| 11 \| Included vitamin B12 in diet \| \| 12 \| Protected hands (e.g. with gloves) when using nang cracker \| \| 13 \| Only used food or medical grade nitrous oxide \| \| 14 \| Other (specify) \| \| 15 \| Don’t know \| \| 16 \| Skip question \| |
| 13 | Ask only if Q12 = 14 | Specify other health risk |  |
| 14 |  | In the past 6 months, have you experienced any of the following symptoms following your use of nitrous oxide which have persisted for at least two weeks and that you had not experienced before you started using nitrous:  (Multiple responses allowed)  READ OUT ALL RESPONSES | Checkbox, required   \| 1 \| Numbness/tingling around your face or mouth \| \| --- \| --- \| \| 2 \| Numbness/tingling in your hands or feet \| \| 3 \| Difficulty walking or with your balance \| \| 4 \| Difficulty with using your hands and fingers, e.g. operating your phone or writing \| \| 0 \| None of the above \| \| 5 \| I don’t know \| \| 6 \| Skip question \| |
|  |  | In the past 6 months, have you experienced a vitamin B12 deficiency (demonstrated with a blood test) that you did not have before you started using nitrous or that has become worse since you started using nitrous? | Radio, required   \| 0 \| No \| \| --- \| --- \| \| 1 \| Yes \| \| 7 \| Don’t know \| \| 8 \| Skip question \| |

***Table S3. Ecstasy and Related Drug Reporting System nitrous oxide module, 2022***

| 917 | h_sectiontext_5  Show the field ONLY if:  [b7_11u6] = 1 | Nitrous Oxide Access | descriptive |
| --- | --- | --- | --- |
| 918 | na1  Show the field ONLY if:  [b7_11u6] = 1 | Nitrous oxide access: The next few questions are about how you have accessed nitrous, and any harms that you may be aware of and/or experienced in relation to use of nitrous oxide. | descriptive |
| 919 | na2  Show the field ONLY if:  [b7_11u6] = 1 | Where did you purchase the nitrous oxide you used in the past 6 months?  FYI - Head shops sell cannabis paraphernalia and items related to countercultures. | checkbox, Required   \| 0 \| na2___0 \| Did not purchase/given/traded \| \| --- \| --- \| --- \| \| 1 \| na2___1 \| Friend/acquaintance \| \| 2 \| na2___2 \| Dealer (not a 24/7 delivery service) \| \| 3 \| na2___3 \| 24/7 delivery service \| \| 4 \| na2___4 \| Corner store/convenience store \| \| 5 \| na2___5 \| Head shops (e.g., Off Ya Tree) \| \| 6 \| na2___6 \| Adult stores \| \| 7 \| na2___7 \| Online shop (but not a 24/7 delivery service) \| \| 8 \| na2___8 \| Hospitality/catering/kitchen shop \| \| 9 \| na2___9 \| Other (specify) \| \| 97 \| na2___97 \| Don't know \| \| 98 \| na2___98 \| Skip question \|   Field Annotation: @NONEOFTHEABOVE='0,97, 98' |
| 920 | na3  Show the field ONLY if:  [na2(9)] = 1 | Specify other source  *997, Don't Know\| 998, Skip question* | text, Required |
| 921 | na13  Show the field ONLY if:  [b7_11u6] = 1 | In the past 6 months, have you experienced any of the following symptoms following your use of nitrous oxide which have persisted for at least two weeks and that you had not experienced before you started using nitrous:  (Multiple responses allowed) READ OUT ALL RESPONSES | checkbox, Required   \| 1 \| na13___1 \| Numbness/tingling around your face or mouth \| \| --- \| --- \| --- \| \| 2 \| na13___2 \| Numbness/tingling in your hands or feet \| \| 3 \| na13___3 \| Difficulty walking or with your balance \| \| 4 \| na13___4 \| Difficulty with using your hands and fingers, e.g. operating your phone or writing \| \| 0 \| na13___0 \| None of the above \| \| 7 \| na13___7 \| Don't know \| \| 8 \| na13___8 \| Skip question \|   Field Annotation: @NONEOFTHEABOVE='0,7, 8' |
| 922 | na14  Show the field ONLY if:  [b7_11u6] = 1 | In the past 6 months, have you experienced a vitamin B12 deficiency (demonstrated with a blood test) that you did not have before you started using nitrous or that has become worse since you started using nitrous? | radio, Required   \| 0 \| No \| \| --- \| --- \| \| 1 \| Yes \| \| 7 \| Don't know \| \| 8 \| Skip question \|   Field Annotation: @NONEOFTHEABOVE='0,7, 8' |
| 923 | na4_1  Show the field ONLY if:  [na2(1)] = 1 or [na2(2)] = 1 or [na2(3)] = 1 or [na2(4)] = 1 or [na2(5)] = 1 or [na2(6)] = 1 or [na2(7)] = 1 or [na2(8)] = 1 or [na2(9)] = 1 | In October 2021 the Therapeutic Goods Administration changed the scheduling of nitrous oxide to try and minimise harms experienced as a result of recreational use. Specifically, nitrous oxide that is supplied in small bulbs, and which is not for therapeutic use, will soon be required to contain a warning statement or safety direction. | descriptive |
| 924 | na15_1  Show the field ONLY if:  [na2(1)] = 1 or [na2(2)] = 1 or [na2(3)] = 1 or [na2(4)] = 1 or [na2(5)] = 1 or [na2(6)] = 1 or [na2(7)] = 1 or [na2(8)] = 1 or [na2(9)] = 1 | The last time you purchased nitrous oxide, were you aware of any warning statements or safety directions on the packaging or individual units (bulbs)? | radio, Required   \| 0 \| No \| \| --- \| --- \| \| 1 \| Yes \| \| 7 \| Don't know \| \| 8 \| Skip question \| |
| 925 | na15_2  Show the field ONLY if:  [na15_1] = 1 | What did the warning or safety directions say? | radio, Required   \| 0 \| Can't remember \| \| --- \| --- \| \| 1 \| Do not intentionally inhale \| \| 2 \| May cause irreversible nerve damage \| \| 3 \| Contact Poisons Information Centre \| \| 4 \| Other (specify) \| \| 7 \| Don't know \| \| 8 \| Skip question \| |
| 926 | na4  Show the field ONLY if:  [na15_2] = 4 | Specify other warning or safety direction  *997, Don't Know\| 998, Skip question* | text, Required |

***Table S4. Ecstasy and Related Drug Reporting System nitrous oxide module, 2023***

| 918 | na1  Show the field ONLY if:  [b7_11u6] = 1 | Nitrous oxide access: The next few questions are about forms of nitrous you have used recently, harms experienced in relation to use of nitrous oxide and awareness of warning labels on products. | descriptive | |  |
| --- | --- | --- | --- | --- | --- |
|  |  | Which form/s of nitrous oxide did you use in the past 6 months?  (Multiple responses allowed)  SHOW FLASHCARD | Checkbox, required   \| 1 \| Small bulbs (~8g) \| \| --- \| --- \| \| 2 \| Bottle/cylinder/tank (≤1000g) \| \| 3 \| Bottle/cylinder/tank (>1000g) \| \| 4 \| Don’t know \| \| 5 \| Skip question \| | |  |
| 919 | na2  Show the field ONLY if:  participant nominated tanks/cylinders | Where did you purchase the nitrous oxide bottle/cylinder/tank/s you used in the past 6 months? Note. This excludes small bulbs.  POINT TO LARGER PRODUCTS FLASHCARD   FYI - Head shops sell cannabis paraphernalia and items related to countercultures. | checkbox, Required   \| 0 \| na2___0 \| Did not purchase/given/traded \| \| --- \| --- \| --- \| \| 1 \| na2___1 \| Friend/acquaintance \| \| 2 \| na2___2 \| Dealer (not a 24/7 delivery service) \| \| 3 \| na2___3 \| 24/7 delivery service \| \| 4 \| na2___4 \| Corner store/convenience store \| \| 5 \| na2___5 \| Head shops (e.g., Off Ya Tree) \| \| 6 \| na2___6 \| Adult stores \| \| 7 \| na2___7 \| Online shop (but not a 24/7 delivery service) \| \| 8 \| na2___8 \| Hospitality/catering/kitchen shop \| \| 9 \| na2___9 \| Other (specify) \| \| 97 \| na2___97 \| Don't know \| \| 98 \| na2___98 \| Skip question \|   Field Annotation: @NONEOFTHEABOVE='0,97, 98' | |  |
| 920 | na3  Show the field ONLY if:  [na2(9)] = 1 | Specify other source  997, Don't Know\| 998, Skip question | text, Required | |  |
| 921 | na13  Show the field ONLY if:  [b7_11u6] = 1 | In the past 6 months, have you experienced any of the following symptoms following your use of nitrous oxide which have persisted for at least two weeks and that you had not experienced before you started using nitrous:  (Multiple responses allowed) READ OUT ALL RESPONSES | checkbox, Required   \| 1 \| na13___1 \| Numbness/tingling around your face or mouth \| \| --- \| --- \| --- \| \| 2 \| na13___2 \| Numbness/tingling in your hands or feet \| \| 3 \| na13___3 \| Difficulty walking or with your balance \| \| 4 \| na13___4 \| Difficulty with using your hands and fingers, e.g. operating your phone or writing \| \| 0 \| na13___0 \| None of the above \| \| 7 \| na13___7 \| Don't know \| \| 8 \| na13___8 \| Skip question \|   Field Annotation: @NONEOFTHEABOVE='0,7, 8' | |  |
|  | Show the field ONLY if:  [na13___1] = 1  [na13___2] = 1  [na13___3] = 1  [na13___4] = 1 | The last time you experienced these symptoms, what treatment(s) did you receive? | checkbox, Required   \| 1 \| e2_1a_5___1 \| Did not receive treatment \| \| --- \| --- \| --- \| \| 2 \| e2_1a_5___2 \| Ambulance attendance \| \| 3 \| e2_1a_5___3 \| Hospital emergency department \| \| 4 \| e2_1a_5___4 \| GP \| \| 10 \| e2_1a_5___10 \| Other (specify) \| \| 97 \| e2_1a_5___97 \| Don't know/can't remember \| \| 98 \| e2_1a_5___98 \| Skip question \|   Field Annotation: @NONEOFTHEABOVE='1,97,98' | | |
|  |  |  | Please specify other treatment | text, Required | |
| 923 | na4_1  Show the field ONLY if:  [na2(1)] = 1 or [na2(2)] = 1 or [na2(3)] = 1 or [na2(4)] = 1 or [na2(5)] = 1 or [na2(6)] = 1 or [na2(7)] = 1 or [na2(8)] = 1 or [na2(9)] = 1 | In October 2021 the Therapeutic Goods Administration changed the scheduling of nitrous oxide to try and minimise harms experienced as a result of recreational use. Specifically, nitrous oxide not supplied for therapeutic use, is now required to contain a warning statement or safety direction. | descriptive | |  |
| 924 | na15_1  Show the field ONLY if:  [na2(1)] = 1 or [na2(2)] = 1 or [na2(3)] = 1 or [na2(4)] = 1 or [na2(5)] = 1 or [na2(6)] = 1 or [na2(7)] = 1 or [na2(8)] = 1 or [na2(9)] = 1 | The last time you purchased nitrous oxide, did you see any warning statements or safety directions on the packaging of individual units (bulbs or cylinder)? | radio, Required   \| 0 \| No \| \| --- \| --- \| \| 1 \| Yes \| \| 7 \| Don't know \| \| 8 \| Skip question \| | |  |
| 925 | na15_2  Show the field ONLY if:  [na15_1] = 1 | What did the warning or safety directions say? | checkbox, Required   \| 0 \| Can't remember \| \| --- \| --- \| \| 1 \| Do not intentionally inhale \| \| 2 \| May cause irreversible nerve damage \| \| 3 \| Contact Poisons Information Centre \| \| 4 \| Other (specify) \| \| 7 \| Don't know \| \| 8 \| Skip question \| | |  |
| 926 | na4  Show the field ONLY if:  [na15_2] = 4 | Specify other warning or safety direction  997, Don't Know\| 998, Skip question | text, Required | |  |
| 927 |  | In the past 6 months, have you been denied sale and/or been unable to access nitrous oxide products? | Checkbox, required   \| 0 \| No, haven’t tried to buy it \| \| --- \| --- \| \| 1 \| No, tried and could buy it \| \| 2 \| Yes, the shop did not stock the product I was after \| \| 3 \| Yes, the seller asked for my ID (and I didn’t have it on me) \| \| 4 \| Yes, the seller said they couldn’t sell it to me because of the time of day/night \| \| 5 \| Yes, the seller told me they couldn’t sell them to me because I was too intoxicated \| \| 6 \| Yes, the seller told me they couldn’t sell them to me for another reason \| \|  \| Yes, the seller refused to sell a particular type or size of product \| \|  \| Yes, the seller imposed a limit on the amount I could purchase \| \| 7 \| Don't know \| \| 8 \| Skip question \| | |  |
|  |  | What was the other reason you were denied sale of nitrous oxide products |  | |  |
|  | com45x  Show the field ONLY if:  [b7_11u6] = 1 | Interviewer comments | text | |  |
